# Investigation of choline-binding protein of CbpD in the pathogenesis of *Streptococcus suis* type 2

**DOI:** 10.3389/fvets.2024.1486347

**Published:** 2024-12-03

**Authors:** Lexin Zhu, Mengqing Li, Guijun Yu, Dongbo Zhan, Wenzhen Zeng, Nanyan Fu, Xiaowu Jiang

**Affiliations:** ^1^College of Medicine, Yichun University, Yichun, China; ^2^Jiangxi Provincial Key Laboratory of Active Component of Natural Drugs, Poster-Doctoral Research Center, Yichun, China

**Keywords:** *Streptococcus suis* type 2, choline-binding protein of CbpD, pathogenicity, virulence factors, modulation

## Abstract

*Streptococcus suis* serotype 2 (*S. suis* type 2, SS2) is one of the zoonotic pathogens known to induce meningitis, septicemia, and arthritis in both pigs and humans, resulting in public health concerns. CbpD, also termed CrfP, is one of the choline-binding proteins (CBPs) that was found as a murein hydrolase in SS2 and plays crucial roles in natural genetic transformation under the control of ComRS-ComX regulatory system by a previous study. Nonetheless, the possible functions of CbpD in virulence and pathogenesis in SS2 remain unclear. In this study, a *cbpD* gene mutant (*ΔcbpD*) with its complemental strain (*cΔcbpD*) was constructed and further used to examine the pathogenic roles of CbpD in SS2 infection. The results showed that the CbpD deficiency leads to increased bacterial chain elongation and aggregation with little impact on the growth capability of SS2. The *ΔcbpD* strain represented more vulnerable to a thermo, acid, or oxidative stress. Elevated adhesion to human epithelial HEp-2 cells, decreased invasion into bEND3.0 cells, and more easily phagocytosed by murine RAW264.7 macrophages of *ΔcbpD* were found. The virulence of *cbpD* mutant was attenuated in a mouse infection model. Enhanced susceptibility within mice blood and impaired ability to colonize organs with alleviated histopathological lesions were also demonstrated as compared with wild-type SS2. It is noteworthy that the discrepant expression of multiple virulence-associated factors including serine/threonine phosphorylase Stp, anti-phagocytosis factor of transglutaminase TGase and adhesin of chaperon DnaJ, were examined resulting from the deletion of *cbpD*. Overall, these findings provided evidence that the CbpD factor contributes to SS2 infection and is involved in bacterial adhesion, invasion, and anti-phagocytosis processes by modulating crucial virulence-associated factors expression.

## 1 Introduction

*Streptococcus suis* (*S. suis*), a globally distributed and underestimated emerging Gram-positive pathogen, is zoonotic and can infect both swine and humans, and lead to symptoms such as septicemia, arthritis, meningitis, and streptococcal toxic shock-like syndrome (1–4). According to the polymorphism among the capsular polysaccharide antigen, *S. suis* has been classified into at least 56 serotypes, i.e., authentic serotypes 1–19, 21, 23–25, 27–31, 1/2, Chz, and 26 novel capsular polysaccharide loci (NCL) (5, 6). *S. suis* serotype 2 (SS2) is the most common and frequently reported isolate, capable of causing significant economic losses in the swine industry and has induced three large-scale outbreaks among humans in China (7–9). Currently, over 100 putative virulence factors of *S. suis* have been reported, including the typical suilysin (Sly), muramidase release protein (MRP), superoxide dismutase SodA, enolase, transglutaminase (TGase), heat shock protein of DnaJ, and serine/threonine kinase (Stk). However, the complex roles of various virulence factors with their potential regulatory networks and molecular interactions with the host are not yet fully understood (2, 10).

Choline-binding proteins (CBPs) are clusters of the important surface-anchored and conservatively distributed proteins in *Streptococcus* (11, 12). In *Streptococcus pneumoniae* (*S. pneumoniae*), the cell wall-associated CBPs typically consist of at least two domains: a highly conserved choline-binding domain (CBD) with characteristic repeat motifs and a functional domain (FD) (13, 14). The CBD functions to bind phosphorylcholine molecules on the cell wall through non-covalent interactions and is essential for microbe interaction with host cells for bacteria colonization in the upper respiratory tract. Fundamental divergence of the three-dimensional structure leading to the physiological discrepancy of FD contributes to CBP varieties (11, 15). To date, CBPs have been reported to be implicated in multiple roles in pneumococcal infection and pathogenesis (16, 17). The conventional CBPs of LytA, LytB, LytC, PspA, PspC, PcpA, Pce, and CbpD within *S. pneumoniae* are found to be an extracellular pneumococcal virulence determinant which can mediate not only for bacterial cell wall construction, colonization to host epithelial cells but also for antibiotics susceptibility and biofilm formation (18–25). Horizontal gene transfer contributes to pathogens’ adaptive evolution and genomic diversity that enables the acquisition of beneficial genes involved in pathogenesis and immune evasion (24, 26, 27). CBPs are also murein hydrolases that are involved in cellular autolysis during the stationary phase and fratricide during the competence process (28–30).

CbpD, one of the well-studied CBPs in *S. pneumoniae*, is characterized by an N-terminal cysteine and a histidine-dependent amidohydrolase/peptidase (CHAP) domain, followed by one or two Src homology 3b (SH3b) domains, and a C-terminal CBD with four choline-binding repeats (26). The CHAP domain is responsible for cleaving peptide bonds within the bridges of streptococcal peptidoglycan, while the SH3b domain plays an essential role in the critical biological function of CbpD, possibly by binding to the peptidoglycan portion of the cell wall. Moreover, CbpD can be involved in inducing virulence factor release from non-competent *S. pneumoniae* upon competent condition (31, 32). Recently, CrfP, a CbpD-like murein hydrolase, first found in *S. suis* type 2, has been identified as a key component in SS2’s competence, enhancing transcription by inducing peptides (33). This study aims to investigate the roles of the underlying pathogenesis of CbpD in SS2 infection.

## 2 Materials and methods

### 2.1 Bacterial agents, plasmids, and culture conditions

Bacterial strains and plasmids utilized for cloning and mutant construction are detailed in Table 1. The SS2 virulent wild-type (WT) strain of HA9801, isolated from diseased pigs which mainly manifested septicemia among SS2 outbreak in Jiangsu, China, was kindly offered by Professor Weihuan Fang at Zhejiang University. The *S. suis* strains were cultured in Brain heart infusion (BHI; Oxoid, UK) at 37°C. *Escherichia coli* DH5α cells were cultured in Luria–Bertani medium at 37°C. The various antibiotics (Solarbio, Beijing, China) used for cloning and the selection of mutants were incorporated into the culture media at specific concentrations: 50 μg/mL of ampicillin for *E. coli*, 100 μg/mL and 50 μg/mL of spectinomycin for SS2 and *E. coli*, respectively.

**Table 1:** Bacterial strains and plasmids used in this study.

Strains or plasmids	Phenotype or features	Source or reference
Bacterial strains		
*S. suis* HA9801	Virulent SS2 wild-type strain isolated from clinically diseased pigs	Preserved in our laboratory
*ΔcbpD*	The *cbpD* gene deletion mutant based on the HA9801	This study
c*ΔcbpD*	The complemental strain of the *cbpD* gene based on the *ΔcbpD*	This study
*E. coli* DH5α	Cloning host for the recombinant plasmids	TransGen Biotech
Plasmids		
pSET4s	Thermosensitive suicide vector for *S. suis* target gene mutation	33
pSET2	*S*huttle vector for *S. suis* mutant gene complementation	33
pSET4s::*cbpD*	Recombinant vector of *cbpD* gene ligated with pSET4s for construction of the *ΔcbpD* mutant	This study
pSET2:: cbpD	Recombinant vector of allelic *cbpD* gene for the construction of c*ΔcbpD* strain	This study

### 2.2 Construction of the *cbpD* mutation and complementation strains

The *cbpD* deletion mutant (*ΔcbpD*) based on wild-type strain HA9801 was constructed as previously described (34). In brief, the upstream and downstream flanking fragments of the *cbpD* gene were amplified by PCR based on the template of the HA9801 genome and the primers listed in Table 2. The flanking fragments were then fused by overlapping PCR. The product was subjected to endonuclease enzymatic digestion, ligated with the thermo-sensitive vector of pSET4s, and then transformed into the DH5α competent cells to generate the knockout plasmid of pSET4s::*cbpD* after PCR identification and DNA sequencing. The recombinant pSET4s::*cbpD* shuttle vector was introduced into HA9801 competent cells via electroporation. The resulting *ΔcbpD* mutant was obtained through a series of screening steps including repeated SS2 passage, targeted gene double exchange, and positive mutant colony isolation, and confirmed by PCR verification and DNA re-sequencing. To construct the allelic *cbpD* complementation strain of c*ΔcbpD*, the previously reported *impdh* promoter was fused with the *cbpD-*ORF and subsequently integrated into pSET2 to yield the complemental plasmid pSET2::*cbpD*. The recombinant plasmid was electroporated into the *ΔcbpD* competent cells and the resulting c*ΔcbpD* isolate was screened by spectinomycin resistance and PCR confirmation.

**Table 2:** Primers used in this study.

Assays	Primer names and sequences (5′-3′)	Function
PCR	cbpD-5’F (*EcoR*I): CCGGAATTCGTCGTTGGTCAGGAGCA	Upstream fragment amplification of *cbpD*
	cbpD-5’R: CTTTATTCATTATTTCTCCTTGTG	
	cbpD-3’F: ATGAATAAAGTTATTACTGCCGAAG	Downstream fragment amplification of *cbpD*
	cbpD-3’R (*Bam*HI): CGCGGATCCAGAGCCAGTAGCGAATCC	
	cbpD-IF: ACCTGCCTATGGTGATGCC	Internal fragment of *cbpD* identification
	cbpD-IR: AGTTGGTTGTTCCCGCTCC	
	cbpD-OF: CGATAAGATGGACGAAGGTA	Outside the homologous region identification
	cbpD-OR: GAAACGGTCAGTCATGCTC	
	SPC-F: GTTCGTGAATACATGTTATA	Spectinomycin gene identification
	SPC-R: GTTTTCTAAAATCTGAT	
	cbpD-F (*Bam*HI): CGCGGATCCATGAAAACATTCTTGAGAAGAAA	CbpD protein expression
	cbpD-R (xhoI): CCGCTCGAGTTAGGAGATGGCGATGTAGC	
	CcbpD-F: AGGAAAGAACATGAAAACATTCTTGAGAAGAA	Complemental gene amplification
	CcbpD-R (*Eco*RI): CTGAATTCTTAGGAGATGGCGATGTAG	
	Pimp-F (*Pst*I): GGCTGCAGATGGAGGCAGGACAGGTAT	impdh promoter amplification
	Pimp-R: ATGTTTTCATGTTCTTTCCTTTCTTTTGGG	
	SS2-16 s-F: CAGTATTTACCGCATGGTAGATAT	SS2 identification
	SS2-16 s-R: GTAAGATACCGTCAAGTGAGAA	
RT-qPCR	q*16S*-F: GTAGTCCACGCCGTAAAC	*16S rRNA* mRNA level for SS2
	q*16S*-R: TAAACCACATGCTCCACC	
	qGAPDH-F: ACGGACCACACCGTGGTGGT	Typical genes’ transcriptional level determination
	qGAPDH-R: GCAGCGTTTACTTCTTCAGCA	
	qenolase-F: GACGTTCGTGATCAACAAGC	
	qenolase-R: CGCAACAGCGATAGAAACAC	
	qsly-F: TGATGAACCAGAATCTCCAAGCAAG	
	qsly-R: GTCTTGATACTCAGCATTGCCACTA	
	qtgase-F: AATCATGTAGTTACGCTCCG	
	qtgase-R: TACAGGGAATAAGCATCAGC	
	qdnaJ-F: GCCAAACCTGGAACAAGTCCG	
	qdnaJ-R: CCGTTACCGTATGGGCTTGTT	
	qstp-F: GAGGCAGTTGTTGTCATCG	
	qstp-R: CAAGGGCACCTACCAG	
	qsod-F: TGGACGGACATTGCGGTAG	
	qsod-R: TCGTTTCGGTTCAGGTTGG	

### 2.3 Growth curves

Growth capability was examined by the protocol instructed previously (35). A single isolated colony of the WT, *ΔcbpD,* and c*ΔcbpD* strains was inoculated into BHI broth for a 12 h incubation at 37°C with 150 rpm shaking. The cultures were centrifuged to form a precipitate and then adjusted to an OD_600nm_ of 0.4 in BHI. Subsequently, 250 μL of the bacterial suspension each was transferred into 25 mL of BHI medium. The cultures were used for spectrophotometric measurement at OD_600nm_ every 2 h after 37°C incubation at 150 rpm. Triplicate assays with three wells each were conducted and determined for this experiment.

### 2.4 Morphological change and stress tolerance analysis

The SS2-WT, *ΔcbpD,* and c*ΔcbpD* strains were Gram-stained on the glass slides following the staining guide and observed by microscopy under oil immersion. The numbers of cocci among these strains in 50 chains each under light microscopy were counted and average bacterial cells per chain were examined. Stress tolerance phenotypes of SS2 strains upon thermo-induced treatment at 42°C, acetic acid treatment at pH5.5 for 37°C or oxidative treatment with 20 mM H_2_O_2_ at 37°C for 1 h, were conducted and examined as previously described, respectively (36).

### 2.5 *In vitro* cell adhesion and invasion assays

The adhesion to and invasion of host cells by SS2 was assessed in accordance with a previous study by using the human laryngeal epithelial HEp-2 cells and the murine brain microvascular endothelial bEND3.0 cells, respectively (36). Briefly, bacterial cultures were added to the cells in a 24-well culture plate at a multiplicity of infection (MOI) of 50, with three wells for each strain. The plates were incubated for 1 h at 37°C, 5% CO_2_. To remove non-adherent bacteria, the wells were washed four times with 1 mL of 10 mM PBS. For the adherence assay, the sterile ddH_2_O was added to lyse infected cells and then plated to BHI agar by 10-fold dilution in PBS. After 24 h incubation at 37°C, the bacteria colonies were enumerated. The adhesion rate was defined as (viable CFU_Adh_ / inoculated CFU_Total_) × 100%. The adhesive inhibition assay was conducted the same as the aforementioned procedure (37), with the exception of the 10% inactivated rabbit polyclonal targeted or pre-immune negative sera treatment of the WT and *ΔcbpD* for 1 h prior to infection.

For the invasion assay, DMEM consisting of gentamicin (300 μg/mL) was introduced into the bacterial infection cells following adhesion assay to eradicate extracellular dissociated SS2 for 1 h at 37°C. The cells underwent subsequently triple washes with PBS and were further lysed by using sterile distilled water. The intracellular evasive bacteria were determined by BHI plate counting. The invasion rate was defined as (viable intracellular CFU_Inv_ / inoculated CFU_Total_) × 100%.

### 2.6 Phagocytosis test

Murine macrophage cell line RAW264.7 was used for the phagocytosis assay according to a previously reported protocol (34, 38). Cells were subcultured and inoculated into 24-well plates at 37°C, 5% CO_2_. The cells were cultivated for 12 h until the cell confluence reached above 90%, washed with PBS, and incubated in DMEM containing 10% inactivated fetal bovine serum. The suspensions of both the WT and *ΔcbpD* strains were pre-adjusted to an OD_600nm_ of 0.4 and then added to the cells at an MOI of 50. After 1 h incubation, the cells were subjected to PBS washing and then treated with gentamicin (300 μg/mL) for another 1 h. Subsequently, the mixture was washed three times by PBS, lysed with sterile distilled water, and plated on BHI agar to count the viable SS2 colonies. The phagocytosis rate was determined by calculating the percentage of surviving CFU in the BHI medium compared to the CFU in the original inoculum. This assay was performed three times for each strain, with triplicate wells tested on each occasion.

### 2.7 Susceptibility in the murine *ex vivo* blood culture

Survival assay of SS2 within mice whole blood was executed following the established protocol (35). Cultures in the mid-exponential growth phase were standardized to an optical density of 0.4 at 600 nm. A 50 μL aliquot of either WT or *ΔcbpD*, was added to 450 μL of fresh whole blood collected from clinically healthy mice and then incubated at 37°C for 1 h. The viable bacterial counts were determined via BHI agar plate enumeration. Each assay was conducted in triplicate and replicated three times.

### 2.8 Virulence determination in mice model

In each designated group, 12 female BALB/c mice, aged 4 weeks, were randomly chosen and subjected to intraperitoneal injection with 1 mL of WT, *ΔcbpD,* and *cΔcbpD* at approximately 5 × 10^8^ CFU/mL, respectively. An equal volume of PBS inoculation served as a negative control. The clinical manifestations and mortality of mice were monitored every 12 h and continuously recorded for 1 week. Furthermore, the heart, liver, spleen, lung, kidney, and brain organs from infected mice showing clinical symptoms were aseptically collected, homogenized, and subsequently subjected to BHI plate counting for bacterial load determination in another SS2 infection experiment. Meanwhile, 4% paraformaldehyde was used to fix tissue samples and then paraffin-embedded sections of the lesion tissues were prepared, stained by Hematoxylin and Eosin, and the histopathological changes of the tissues were examined under a microscope as described previously (37). Animal infection experiments conformed to the animal ethics guidelines from the Medical Ethics Committee of Yichun University in Yichun City, Jiangxi Province, China (2022018).

### 2.9 Quantitative real-time PCR (qPCR) analysis

Bacterial cultures, upon reaching the mid-logarithmic growth phase, were harvested. The cells were then pelleted through centrifugation at 5,000 rpm for 10 min. Total RNA extraction from the bacterial pellets was carried out following the manufacturer’s protocol by the RNA Extraction Kit (TransGen, China) (39). Subsequently, synthesis and amplification of cDNA were performed via a reverse transcription kit (Vazyme, China). To quantify the transcriptional levels of the target genes listed in Table 2, relative qPCR was conducted using the ChamQ Universal SYBR qPCR Master Mix (Vazyme, China) with Bio-Rad CFX96 thermocycler. The *16S rRNA* gene was determined as an internal reference to normalize the gene expression data.

### 2.10 Western blotting

The effect of CbpD deficiency on the virulence-related factors expression levels was analyzed by Western blotting. Overnight cultures of WT and *ΔcbpD* strains were centrifuged at 5,000 rpm for 10 min to collect the bacteria suspension and supernatants. Total protein samples from bacteria suspension, except for two secreted proteins Sly and TGase, which were directly determined from the harvested bacteria supernatant, were utilized for the analysis of protein expression changes. The total SS2 cellular proteins were extracted by cell wall cleavage buffer including 3 mg/mL of lysozyme in 37°C for 1.5 h and homogenizing the mixture following the previously established protocol, and the protein concentration was quantified by the Bradford method (40). SDS-PAGE was performed, followed by protein transfer onto PVDF membranes, blocking, and incubation with rabbit polyclonal antibodies (prepared in-house) at 4°C for 12 h. Then, the PVDF membranes underwent incubation of HRP-conjugated goat anti-rabbit monoclonal IgG, washed five times with TBST containing 0.5% skimmed milk powder, and visualized by chemiluminescent imaging system after a chemiluminescence reaction kit (GLPBIO, United States). Protein changes in grayscale were accomplished via ImageJ software (ImageJ 1.5, NIH, United States).

### 2.11 Statistical analysis

Each experimental assay was performed in triplicate and repeated on at least three distinct occasions, and the resulting data were presented as mean ± standard deviations (SDs). Statistical significance was determined using a paired, two-tailed Student’s t-test, facilitated by the GraphPad Prism 5.0 software. For animal infection experiments, the log-rank test was used to assess the differences in survival rate. The *p*-value threshold of 0.05 was established and considered as the criterion for statistical significance.

## 3 Results

### 3.1 The *cbpD* gene-deficient with its complementary strain construction in SS2

**Figure 1:**
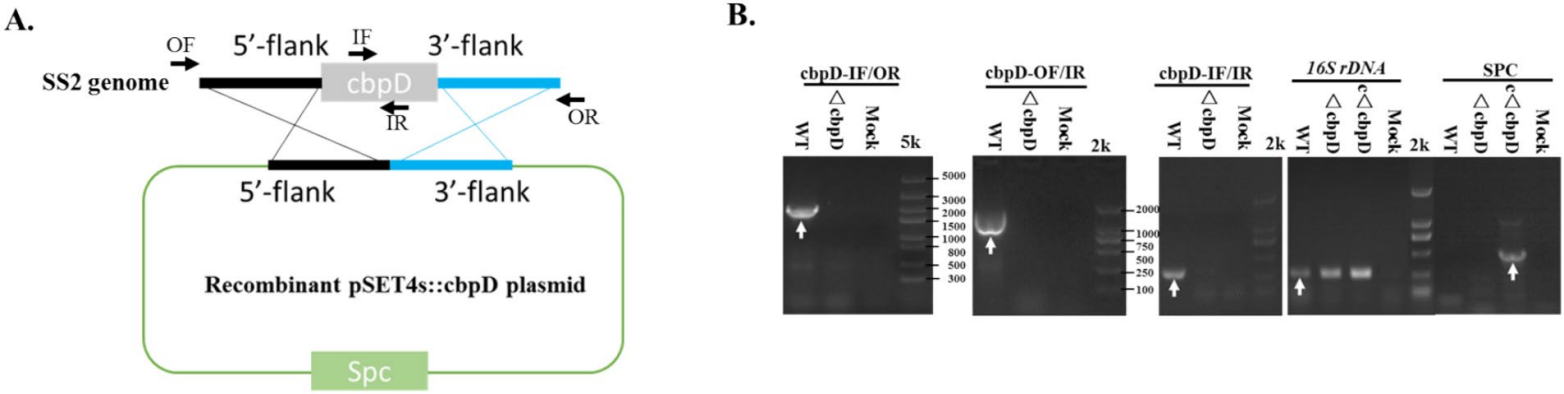
*CbpD*-deficient and complementary strain generation in *S. suis* type 2. **(A)** Schematic diagram for the recombinant pSET4s::cbpD construction. **(B)** PCR identification of the *cbpD* gene deletion and the complementation strains. OF/OR primers and IF/IR primers represented the outer-flank and inner-gene amplification regions for cbpD and were used for gene deletion identification. Antimicrobial resistance gene primers of SPC-F/R were used for cbpD-deficient and complementary identification. The 16S rDNA was used for SS2 identification.

The construction of the *cbpD* mutant and complementation strains was performed using homologous recombination with the shuttle vectors pSET4s for the mutant and pSET2 for the complementation strain. The specific gene knockout plasmid of recombinant pSET4s::*cbpD* was generated as shown in Figure 1A. After electroporation, repeated subculture, and screening, the suspected *ΔcbpD* mutant and allelic c*ΔcbpD* colonies were identified by PCR using the outer-, inner-, and *S. suis*-specific 16S rRNA gene primers, and subjected to verification by DNA sequencing. The *cbpD* mutant with its allelic complementary strain of c*ΔcbpD* was successfully obtained, as evidenced by PCR identification (Figure 1B).

### 3.2 Biological phenotypic changes for the *cbpD* gene deficiency

**Figure 2:**
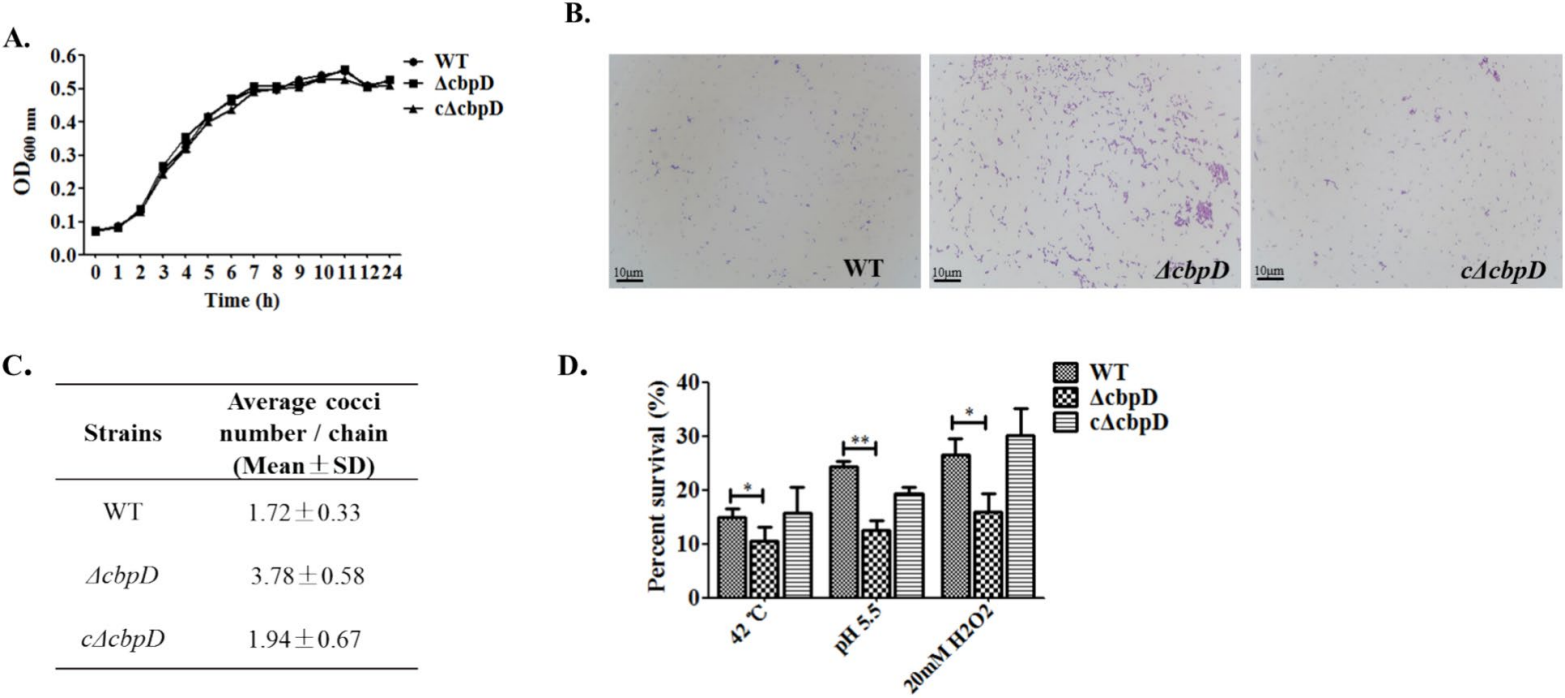
Phenotypic changes for the *cbpD* gene deficiency. **(A)** Growth characteristic among WT, *ΔcbpD,* and c*ΔcbpD* strains. **(B)** Morphological observation under microscopy with a magnification of ×1,000. **(C)** Average bacteria cells in each chain between the WT and *ΔcbpD*. **(D)** SS2 survival assays at different stress conditions.

Figure 2A demonstrated that the growth patterns between the WT and *ΔcbpD* strains were predominantly congruent, and the ablation of *cbpD* does not impinge upon the growth competence of SS2. Morphological observations disclosed that in contrast to the WT strain, the *ΔcbpD* possessed enhanced chain-forming capability, with an average of 3–5 bacterial cells per chain, and manifested a propensity to aggregate (Figures 2B,C). The stress tolerance analysis discovered that the deletion of the *cbpD* gene would significantly decrease the survival rate of *ΔcbpD* and render this strain more susceptible to cultures at 42°C, pH5.5, or 20 mM H_2_O_2_ stress conditions (Figure 2D). The abovementioned deviations could be restored in phenotype after *cbpD* replenishment in c*ΔcbpD* strain.

### 3.3 The CbpD factor of SS2 is engaged in the host cell infection *in vitro*

**Figure 3:**
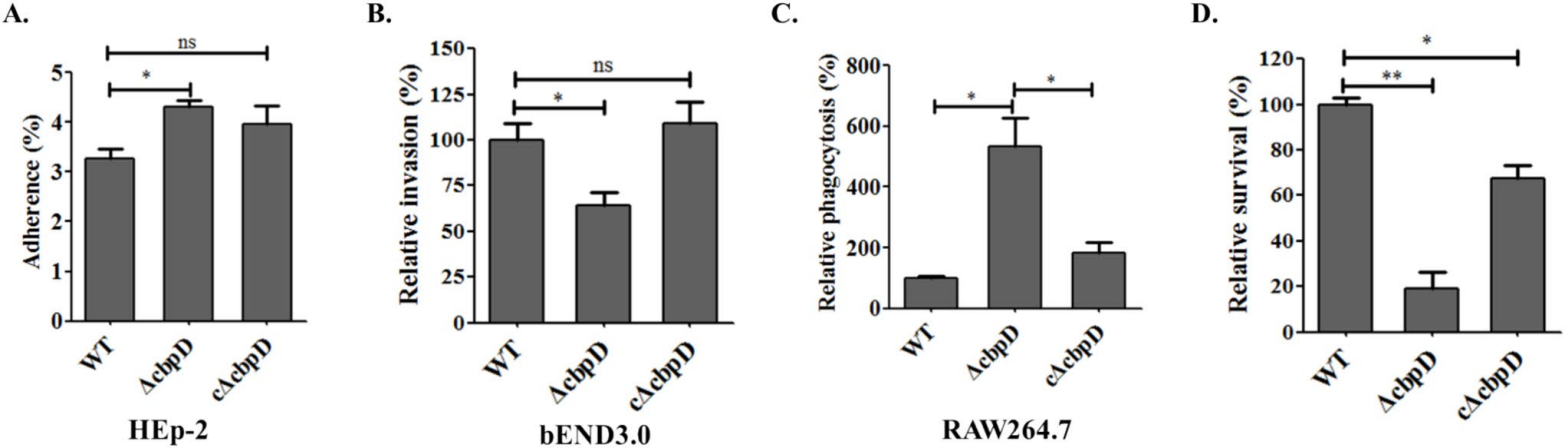
Roles of the CbpD factor among host cell infection. **(A)** Adhesion assay in HEp-2 cells. **(B)** Invasion to bEND.3 cells. **(C)** Phagocytosis rate in murine macrophage RAW264.7. **(D)** Survival capability in mice whole blood. Data were shown as mean ± SD of three independent experiments and the significant differences indicated as *, *p* < 0.05; **, *p* < 0.01.

To examine the potential role of CbpD in SS2 infection, *in vitro* cell infection models previously developed were used to investigate the differences in adhesion, invasion, resistance to phagocytosis, and survival capability among WT, *ΔcbpD,* and c*ΔcbpD* strains. The findings revealed a prominent increase in the adhesion rate of the *cbpD*-deficient strain to HEp-2 epithelial cells (*p* < 0.05, Figure 3A), while the colonies of *ΔcbpD* invading endothelial bEND3.0 cells were notably decreased (*p* < 0.05, Figure 3B). These *cbpD*-deficient SS2 were prone to be phagocytosed by macrophage cells of RAW264.7 and showed an 80% reduction in survival when incubated in *ex vivo* mouse whole blood (*p* < 0.01, Figures 3C,D). The introduction of the *cbpD* gene could partially restore these phenotypic changes. These data implied that the CbpD factor was involved in SS2 infection by mediating the immune invasion and phagocytosis process.

### 3.4 The CbpD is a potential virulence factor involved in the pathogenicity of SS2

**Figure 4:**
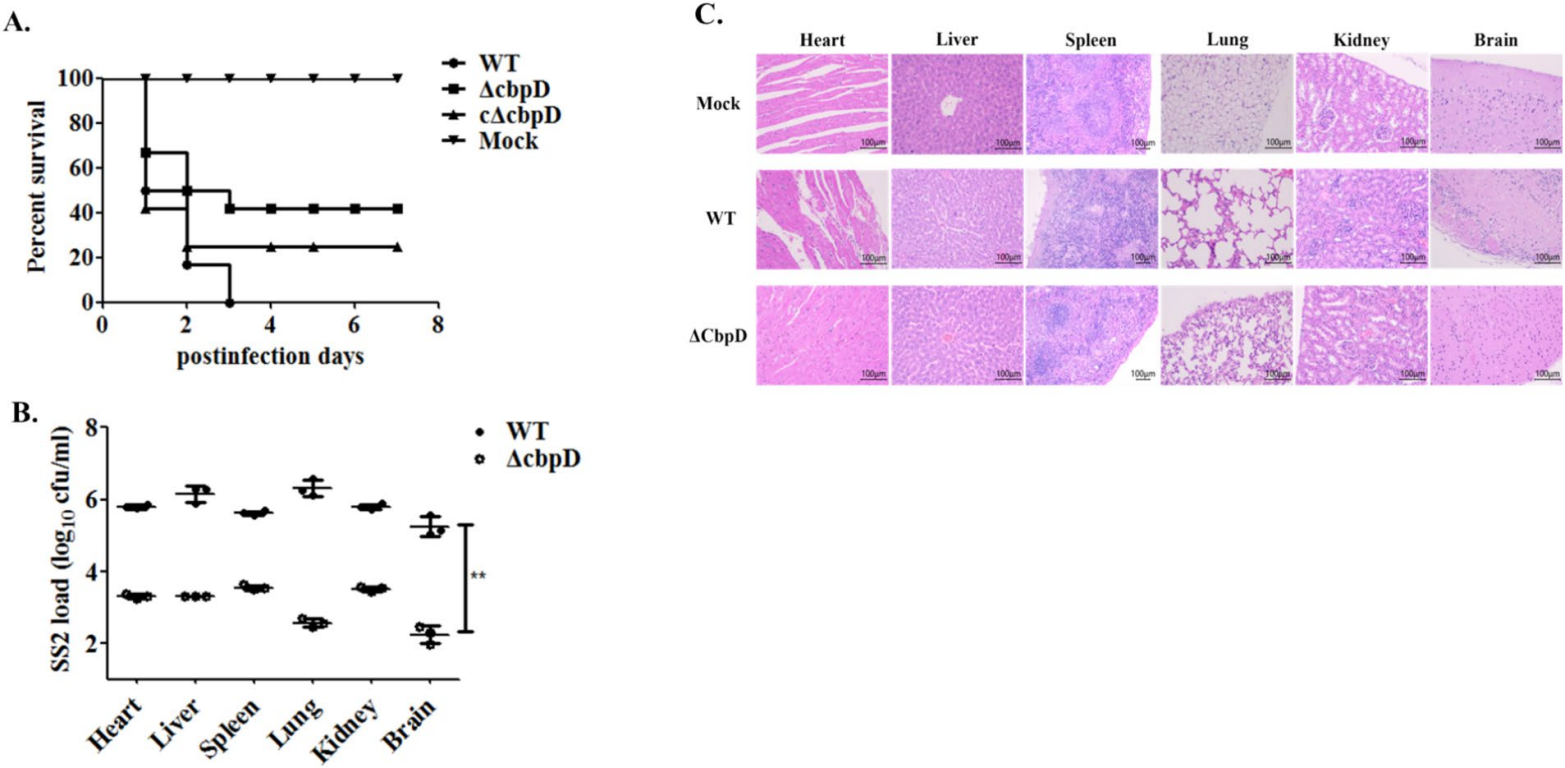
Animal infection model for virulence evaluation. **(A)** Survival curve of the mice challenged with WT, *cbpD* gene mutant, and its complemental strains. **(B)** Bacterial burden in mice organs. Significant difference of ** is indicated as *p*-value of < 0.01. **(C)** Histopathological observation of mice organs infected with the WT, *cbpD* gene mutant and its complemental strain.

An *in vivo* mouse infection model was used to evaluate the impact of *cbpD* gene deletion on SS2 virulence at a lethal dose. The findings indicated that 50% of the mice in the WT infection group died within 24 h, and all died within 72 h. The clinical symptoms in the WT-infected mice were noticeable, with common manifestations such as distinguished disheveled hair, tearing and blindness, mental signs, and movement disorders. Nevertheless, a significant decrease in the virulence of the *cbpD* deletion strain was shown as none of the mice died 3 days after infection, resulting in a survival rate of 41.67% as well as strikingly alleviated clinical symptoms (Figure 4A). The results of bacterial load in mouse organs affirmed that the susceptible strain of the *ΔcbpD* was easier to clear since the prominently reduced colonization colonies of the *ΔcbpD* were shown in mice organs in Figure 4B. In accordance with the results of bacterial burden in organs, the milder and relieved pathological damages, such as myolysis and disruption of cardiac muscle fibers in the heart, hepatocellular necrosis, and inflammatory cell infiltration with hemorrhage in the liver, disintegration of the splenic nodules along with bleeding in the spleen, congestion, and rupture of the alveoli accompanied by hemorrhage in the lung, glomerular enlargement, and infiltration with inflammatory cells in the kidney, and purulent meningitis, neuronal cell swelling and degeneration in the brain in the WT infection group, were induced by the CbpD deficiency SS2 infection based on the microscopic histopathological observation (Figure 4C). The results illustrated that *cbpD* deletion attenuated the virulence of *S. suis 2*.

### 3.5 The CbpD deficiency alters the expression of virulence-related factors in SS2

**Figure 5:**
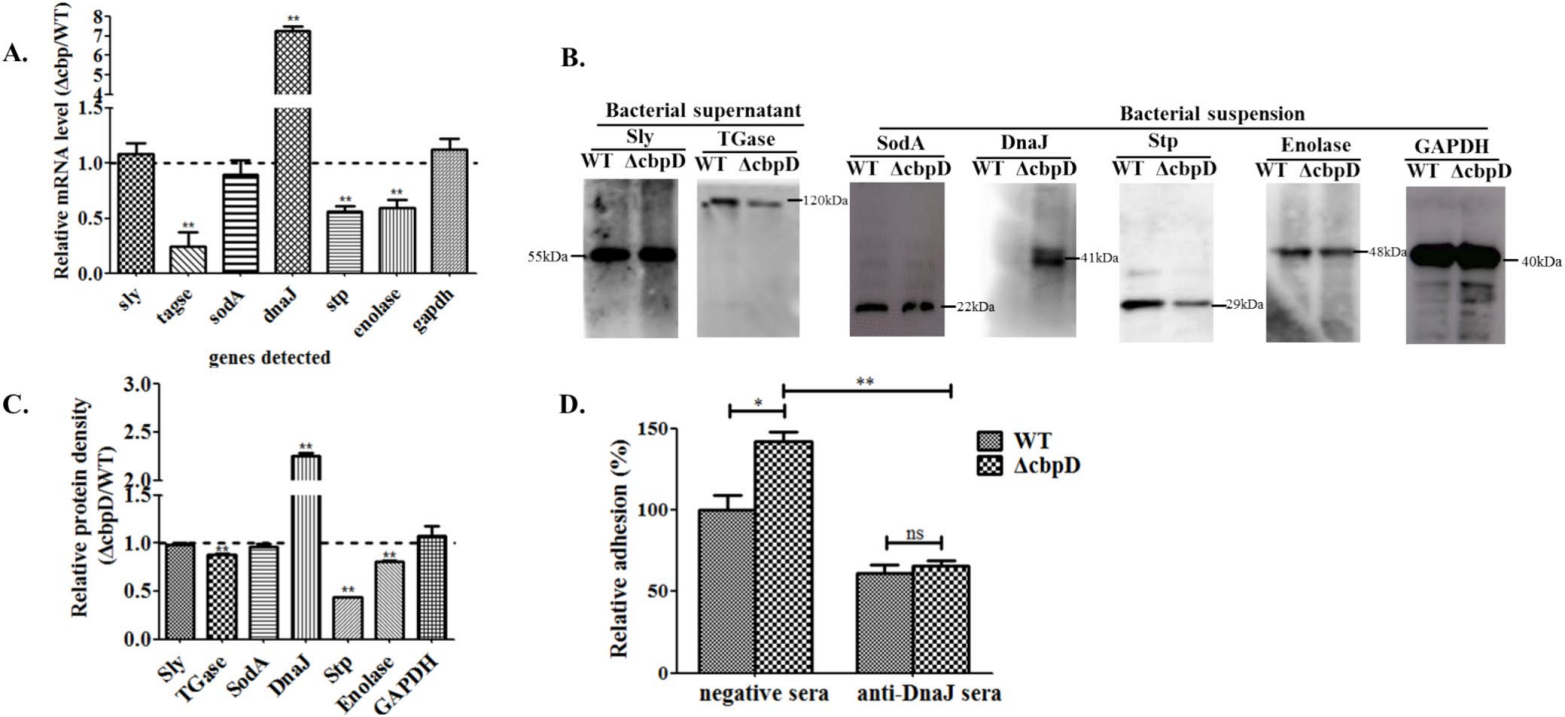
Distinctive virulence-associated factors expression of SS2 in the CbpD factor mutant strain. **(A)** Relative mRNA fold changes for virulence-associated genes of *ΔcbpD* mutant in relation to the WT strain. Significant difference of ** is indicated as *p* < 0.01. **(B)** Western blotting analysis of the correspondingly detected genes in **(A)**. Two secreted proteins of Sly and TGase, and total proteins of SodA, DnaJ, Stp, enolase, and GAPDH were examined from bacteria supernatant and suspension, respectively. **(C)** The densitometric difference analysis for Western blotting result. **(D)** Adhesion inhibition assay for the WT and *ΔcbpD* mutant strains pretreated with antisera against DnaJ. Significant differences of * indicated as *p* < 0.05 and ** as *p* < 0.01.

To dissect the impact of the *cbpD* mutation on bacterial virulence factors expression, seven previously reported virulence-associated factors including the Sly, TGase, SodA, adhesion factor of chaperone DnaJ, Stp, membrane-associated protein of enolase, and glyceraldehyde-3-phosphate dehydrogenase (GAPDH), were examined by transcriptional qPCR and Western blotting analysis. The qPCR results indicated that the deletion of the *cbpD* gene induces the upregulated expression of *dnaJ* and the conspicuously downregulated expression of three genes of *tgase, enolase,* and *stp* (Figure 5A). Further protein level detection by Western blotting proved the corresponding differential alterations of these targeted factors in transcriptional levels (Figures 5B,C).

The adhesive inhibition assay was executed to investigate whether the elevated expression of the adhesion factor of DnaJ in *ΔcbpD* strain prompted the augmented bacterial adherence to HEp-2 cells. The surprising results in Figure 5D revealed that pre-blocked treatment of the *ΔcbpD* mutant with monoclonal DnaJ antisera significantly attenuated its adhesion to HEp-2 cells when compared to the negative pre-immune antisera treatment group (*p* < 0.01).

## 4 Discussion

CbpD is one of the choline-binding proteins, which are major surface-anchored components that are involved in the microbe’s physiological processes and engagement with host cells. CbpD plays a role in the growth, autolysis, and biofilm formation, they are important for the pathogenicity of *S. pneumoniae* (11, 12, 20). Both in *S. thermophilus* and *S. mutants*, CbpD exerts a pivotal function in fratricide as a murein hydrolase and deletion of the CbpD factor would reduce the transformability (12, 29). A recent investigation has confirmed the presence of CbpD in SS2 and its involvement in enhancing transcription by stimulating peptides that expedite the process of transformation (33). Nevertheless, the specific contribution of CbpD to SS2 pathogenicity remains unknown. In this study, a *cbpD* gene knockout mutant and its allelic complementation strain based on the lethal zoonotic strain of HA9801 were obtained by typical homologous recombination method and utilized to explore the prospective phenotypes changes and pathogeneses induced by the CbpD factor in SS2 (Figure 1). As a result, the data unveiled that the *cbpD* deletion mutant exhibited a tendency toward aggregation and a noticeable increase in chain length, and decreased resistance to thermal, acid, and oxidative stress, though no visible growth capability variation was found (Figure 2). Similar morphological abnormality such as cell elongation and septum misplacement was examined and verified in *S. pneumoniae* (41). This implied the probable impact of CbpD on bacterial cell division and stress tolerance and required further investigation.

Stable adherence to host cells constitutes a crucial step in pathogenic microbe colonization and invasion (42, 43). Zoonotic *S. suis* strains primarily colonize through the bodies’ respiratory epithelial tissue and the virulent strain possesses the capacity to release a variety of virulence factors, such as suilysin, to compromise the integrity of the epithelial cells and invade the bloodstream (3, 44). They propagate and multiply through immune evasion and other sophisticated modalities (10). Moreover, these virulent strains have the potential to transgress the blood–brain barrier and thereby instigate the occurrence of meningitis (45). In the current study, the inactivation of CbpD was found to enhance the SS2 adherence to the HEp-2 cells and reduce their capability of invasion into bEND.3 cells (Figure 3). High adhesion indicated a stronger colonization for SS2, which may be related to the differential expression of adhesins. Similar results have been found in *prsA* gene mutant and vaccine candidate strain of SS2 (34, 37), while the invasiveness of pathogens which can destroy the host immune barrier is closely related to virulence factors penetration. Additionally, it was noted that the deletion of *cbpD* rendered SS2 unable to induce a systemic infection as shown by lower lethality compared to the WT strain in BALB/c mice (Figure 4). The CbpD deficiency resulted in an increased phagocytosed SS2 by macrophage RAW264.7 and a significantly reduced survival capability in mice whole blood (Figures 3, 4). A higher phagocytosis rate owing to the CbpL deficiency in *S. pneumoniae* was investigated (46). Notably, mice infected with *ΔcbpD* mutant exhibited a significant reduction in bacterial loads within organs compared to those infected with the WT strain and an effectively mitigated pathological lesion (Figure 4). These findings indicated that CbpD plays a crucial role for SS2 in counteracting host-killing mechanisms by potentially regulating the adhesion process and contributing to anti-phagocytosis as part of its immune evasion response to the host.

Microorganisms encounter diverse microenvironments during the process of host infection, and they can respond to stimuli by modulating the production of proteins essential for a specific process (47). It is well known that the pathogenicity of SS2 demands the complicated and coordinated action of a multitude of virulence-associated factors (42, 45, 48). GAPDH and Enolase have been found to act as crucial adhesins for *S. suis*, which bind to the extracellular matrix of the host and facilitate adhesion (42, 43). Stp, one of the Ser/thr protein phosphatase, is verified to participate in SS2 infection by regulating capsule thickness and virulence factors translocation (49). TGase is an important virulence factor which can mediate the crucial pathogenic process of antiphagocytosis (50). Sly is also instrumental in anti-phagocytic processes, facilitating bacterial evasion of phagocytosis, countering phagocytosis, and ensuring intracellular viability (44). DnaJ, a pivotal element situated within the DnaK chaperone molecular operon, was identified to act as an adhesin factor contributing to cell adhesion and thermotolerance in SS2 (40). The main function of SodA is to catalyze the superoxide ions released by the respiratory bursts in phagocytes, thus affecting pathogenicity (51). The qRT-PCR analysis and Western blotting confirmation results revealed that CbpD factor deficiency could lead to the discrepant alteration of virulence-associated gene expression. The proteins of enolase, Stp, and the anti-phagocytosis factor of TGase, were discovered to be downregulated, whereas the adhesin of the DnaJ factor was conspicuously induced to high expression, in the *cbpD* gene mutant strain (Figure 5). Furthermore, the adhesion inhibition test further verified the result of a prominent adhesion rate in *ΔcbpD* strain owing to its enhanced expression of DnaJ, and these data prompt us to suggest that the differential expression of the corresponding virulence-associated factors possibly contribute to the *cbpD* gene-deficient strain being more susceptible to phagocytic clearance and a significant attenuation in pathogenicity.

In conclusion, this study demonstrated the crucial functions of CbpD with its underlying pathogenesis in SS2 infection. However, further validation is still required to ascertain whether this regulation is direct or indirect and to explore the underlying molecular mechanism of the CbpD involved in the pathogenesis of SS2. Moreover, the exploration of the anti-virulence strategies or treatments for SS2 infection, such as developing putative antagonists for the targeted CbpD or CbpD-associated virulence proteins, deserves to be studied in the future.

## Data Availability

The original contributions presented in the study are included in the article/supplementary material, further inquiries can be directed to the corresponding author.
